# Co-occurrence of symptoms of substance and behavioral addictions over time: A secondary analysis of longitudinal data from the Cohort Study on Substance Use Risk Factors

**DOI:** 10.1556/2006.2025.00088

**Published:** 2025-11-24

**Authors:** Andrea Zagaria, Guyonne Rogier, Gerhard Gmel, Simone Amendola

**Affiliations:** 1 Department of Systems Medicine, Tor Vergata University of Rome, Rome, Italy; 2 Saint Camillus International University of Health Sciences, Rome, Italy; 3 Lausanne University Hospital and University of Lausanne, Lausanne, Switzerland; 4 Independent Researcher, Italy

**Keywords:** addictive disorder, transition, longitudinal, development, behavioral addictions

## Abstract

**Background and aims:**

The transdiagnostic Syndrome Model of Addiction considers addiction as a syndrome with multiple opportunities for expression, i.e., a common underlying disorder that may manifest in distinctive ways. Considering that testing of the model has been rare, this study examined the co-occurrence of symptoms of substance use disorders and behavioral addictions over 10 years by identifying profiles and predictors associated with group membership.

**Methods:**

Longitudinal data (*N* = 4,760 males) from the Cohort Study on Substance Use Risk Factors (C-SURF) conducted in Switzerland were analyzed using a latent transition analysis (LTA). Symptoms of substance use (alcohol, cannabis, and tobacco) disorders and behavioral addictions (gaming and gambling) were used for identifying addiction profiles and group membership trajectories. A multinomial logistic regression model examined predictors of profile membership, while two general linear models tested whether profile membership predicted psychosocial outcomes.

**Results:**

LTA revealed three distinct profiles of symptoms of addiction, labelled Low- (91–92% of the sample), Medium- (5–7%), and High-symptom (1–3%) profiles. Thus, frequency or intensity of addiction symptoms tended to co-occur. The stability of Low-profile membership remained consistently high over time (97%), whereas the stability of Medium- and High-profile memberships was moderate (40–49%). Sensation seeking, neuroticism, parental attitude, and poor parental monitoring were associated with Medium and High profiles compared to the Low profile. Poor relationships with parents and friends were uniquely associated with High profile, whereas hostility, sociability, and family history of mental disorders were associated with Medium profile. Education, sensation seeking, hostility, and neuroticism were associated with transitions between profiles over time. Profile membership predicted subsequent life satisfaction and negative life events.

**Discussion and conclusions:**

Symptoms of substance use disorders and behavioral addictions tend to co-occur, and profile membership is relatively stable. However, transition to less severe profiles also occurs and is influenced by education and personality traits. Our findings have implications for preventive interventions aiming at reducing the risk of addictive disorders becoming chronic and for identifying young adults most in need of support.

## Introduction

### The Syndrome Model of Addiction

Shaffer et al.'s (2004) transdiagnostic Syndrome Model of Addiction proposes to consider addiction as a syndrome with multiple opportunities for expression (e.g., gambling, substance abuse). There are many similarities between the different expressions of addiction, which reflect a common etiology, a syndrome (Shaffer et al., 2004). The antecedents of the addiction syndrome include different levels of individual vulnerability (e.g., psychosocial, neurobiological), exposure to the object, and interactions with it. According to the authors, a requirement for the development of addiction is the encounter with an object that “fits” with the neurobiological and psychosocial antecedents, repeatedly producing a desirable and sought-after subjective state representing the emergence of the premorbid stage of addiction. Thus, “addiction is not necessarily inextricably linked to a particular substance or behavior” (p. 370) (Shaffer et al., 2004). In this context, the Syndrome Model of Addiction represents a useful theoretical perspective to understand the potential existence and validity of a multitude of behavioral addictions (Brand et al., 2020), as behavioral addictive disorders might be better understood considering their common etiology, antecedents, and manifestations rather than specific symptoms. In a similar fashion, a recent contribution emphasized the centrality of dysfunction in impaired control in addictive disorders (Amendola, Hengartner, & Wakefield, 2025).

The proposal of the transdiagnostic syndrome model of addiction has received substantial attention from the scientific community, with more than 474 citations on Scopus as of September 7th, 2025. A prior study tested the validity of the model by examining treatment-seeking groups with substance use addictions and behavioral addictions compared to a group seeking treatment unrelated to addiction (Shaffer et al., 2018). The two addiction groups showed higher trait anxiety and depression, lower emotional support, and higher venting as psychological coping, higher difficulty in identifying feelings, and attentional impulsiveness than the comparison group with no addiction (Shaffer et al., 2018). At the same time, some differences emerged between the substance and behavioral addiction groups, with the latter showing higher state anxiety and self-distraction as psychological mechanisms compared to the former, more inclined to substance use (Shaffer et al., 2018). However, empirical investigations have been scarce to date.

### Cross-sectional studies exploring addiction groups/profiles

Findings from previous studies employing latent group analyses supported the conceptualization of addiction as representing a common underlying disorder that may manifest in distinctive ways. Deleuze et al. (2015) differentiated a group of individuals involved with both substances and behaviors potentially addictive and a second group reporting a comparable or higher presence of behaviors but a lower frequency of substance use. However, latent class analysis was performed using the presence (“at least once per month”) or absence of the behaviors as indicators, rather than addiction scores, and thus may not be directly informative of a syndrome model of addiction. Despite this, subsequent research showed the validity of those findings if symptoms of addiction were considered. Burleigh et al. (2022) examined different profiles of substance use and behavioral addiction symptoms as well as personality traits and coping strategies in three adult samples from the UK, New Zealand, and Australia. In each sample, the authors found a “high-risk” group characterized by high symptoms of both substance use and behavioral addiction, and an “at-risk” group showing high symptoms of behavioral addictions but average substance use. These two groups showed a similar pattern of personality and coping scores across samples, that is, dysfunctional coping and low conscientiousness, but differences with the low-risk group were not tested.

Marmet, Studer, Rougemont-Bücking, and Gmel (2018) examined latent profiles of family background, personality, and mental health factors and their association with behavioral addictions and substance use disorders in young Swiss men using data from the Cohort Study on Substance Use Risk Factors (C-SURF). Symptoms of behavioral addictions and substance use disorders showed similar scores within each latent profile, and this finding led the authors to conclude that “addictions studied share common vulnerabilities that may not be specific to SUD *[substance use disorders]* or BA *[behavioral addictions]*, but rather to addiction in general. This is in line with the addiction syndrome concept” (p. 79) (Marmet et al., 2018).

Finally, in a qualitative study, Kim, Hodgins, Kim, and Wild (2020) investigated whether addictive disorders could be conceptualized from a transdiagnostic perspective by examining the most important indicators of ten addictive behaviors from the perspective of people with lived experiences. According to the authors, the results provide preliminary evidence consistent with the syndrome model of addiction with dependence (e.g., craving, impairments in control) and patterns of use (e.g., frequency) being the most perceived indicators for both substance and behavioral addictions.

### Longitudinal studies, stability, and “hopping” between addiction groups/profiles

In their review of the literature, Shaffer et al. (2004) advocated for the need to test crucial understudied aspects of the syndrome model, such as temporal patterns of association, that is, “hopping between chemical and behavioral addictions”, and temporal patterns of psychiatric comorbidity (such as sign, symptom, and disorder patterns). To date, only a few longitudinal studies have examined the co-occurrence of substance and behavioral addictions despite their relevance in informing preventive (e.g., the identification of persons at higher risk of addictive disorders) and treatment strategies (e.g., focus on key predisposing and maintaining factors). Sussman, Pokhrel, Sun, Rohrbach, and Spruijt-Metz (2015) identified two groups of participants based on their symptoms of eleven addictions: a non-addiction group and an addiction group. Importantly, stability in group membership at one-year follow-up was high (86–90%). In a longitudinal study by Kim, Tabri, and Hodgins (2024), symptoms of gambling addiction and other substance/addictive behaviors were likely to decrease over five years simultaneously. However, to increase variance, the authors used a composite score of substance/behavioral addiction severity (i.e., the severity of substance use and behavioral addictions was combined) rather than distinct substance use and behavioral addiction scores.

### The study objectives

Considering all the above, with the present study, we aimed to expand scientific knowledge on the longitudinal co-occurrence of substance use and behavioral addictions as well as predictors of group membership, using longitudinal data from the C-SURF study. Specifically:

I. We tested whether the pattern of occurrence of substance use and addictive behaviors over time provided support for the syndrome model of addiction (Shaffer et al., 2004) as suggested by a) the identification of profiles showing differences based on the severity of addictive symptoms across substances and behaviors; by b) differential co-occurrence (presence versus absence) of addictive symptoms with no difference in personality traits; and by c) group membership stability over time or (proportion in) shifting from one addiction group to another.
II. We examined whether sociodemographic and personality traits (Shaffer et al., 2018) and family-related variables constituted common risk factors for addiction group membership and trajectory.
III. Finally, we analyzed the association between group membership and trajectory with satisfaction with life, and negative life events at the last follow-up.

## Methods

### Study design and procedures

The present study used data from the Cohort Study on Substance Use Risk Factors (C-SURF) (https://www.c-surf.ch), a longitudinal study designed to investigate substance use patterns and their related consequences in young Swiss men who had to go through the mandatory recruitment process at the Swiss army in 2010–2011 (time 1, T1, baseline). All young men at the recruitment centers in Lausanne (French-speaking), Windisch, and Mels (German-speaking) were invited to participate in the study. These three centers cover 21 of the 26 Swiss cantons, including all French-speaking cantons. As detailed elsewhere (e.g., 11), Switzerland has a mandatory army recruitment process for men and thus the eligible sample was highly representative (almost a complete census) of the Swiss male population. Women can join the army on a voluntary basis, but there are only a few. A female sample drawn from the army would not be representative of the female population in those cantons. Budget constraints did not allow for drawing a costly representative sample of the female population at the age of 20 years outside the army.

Recruitment centers were used only to enroll participants. Questionnaires were sent to participants' homes and confidentiality towards the army was guaranteed. The administered questionnaires were about participants' socio-professional and family background, substance consumption, behavioral addictions, and personality characteristics. Participation was rewarded with a gift voucher shortly after the questionnaires were filled in. The battery of questionnaires was filled in online or using paper and pencil in approximately 45–60 min. The second questionnaire administration (T2, first follow-up) occurred in 2012–2013 about 18 months after the baseline evaluation (T1). The third (T3, second follow-up) and fourth (T4, third follow-up) data collection occurred three years after the previous one in 2016–2017 and 2019–2020, respectively.

Personal information and answers to the questionnaires were highly confidential at all stages and secured under an anonymous coding system.

### Participants

1,569 men out of a total of 7,563 who initially provided written consent to participate in the study did not participate in the first wave. Thus, 4,760 (79.4%) men out of 5,994 completed all four waves of data collection and were therefore included in the present analyses. Attrition analyses revealed small or negligible differences in age and baseline substance and behavioral addiction symptoms between participants retained in the study and those lost to follow-up (i.e., Cohen's *d* < |0.5|; Cohen, 1988). The mean age of participants at baseline was 19.96 years (SD = 1.22; range: 18–28).

### Measures

#### Substance and behavioral addictions

Symptoms of alcohol use disorder during the last 12 months were measured using 12 items based on DSM-IV diagnostic criteria (Grant et al., 2003; Knight et al., 2002) in a yes/no format. Symptoms of a problematic pattern of cannabis use during the last 12 months were examined using the revised version of the Cannabis Use Disorder Identification Test, constituted of 10 items (Annaheim, Scotto, & Gmel, 2010). Symptoms of tobacco dependence during the last 12 months were investigated using the 6-item Fagerström Test for Nicotine Dependence (Heatherton, Kozlowski, Frecker, & Fagerstrom, 1991). Considering that only former drinkers and smokers in the past 12 months completed the above questionnaires exploring symptoms of addiction, we assigned to non-drinkers and non-smokers the lowest possible value for each questionnaire. Symptoms of videogaming addiction during the last 6 months were measured using the 7-item Game Addiction Scale (Lemmens, Valkenburg, & Peter, 2009) (except for the use of an adapted version covering both gaming and internet use at the first two survey waves, i.e., baseline and first follow-up) and based on the diagnostic criteria for pathological gambling reported in the DSM-IV. Finally, symptoms of problematic gambling were assessed by combining two measures exploring gambling behaviors (7 items adapted from the Swiss Health Survey) and self-reported personal problems caused by gambling (self-developed item) during the last 12 months. To combine the two measures, all gambling-related items were rescaled to a 0–1 range using a monotonic proportion of maximum scoring (POMS) transformation (Little, 2013). After being converted to a comparable metric, items were summed to compute a composite score of problematic gambling symptoms.

#### Predictors

##### Family factors

Financial situation of parents was measured asking participants “How well off is your family compared to other families in your country?” with responses distributed on a 7-point Likert scale from “1” (Very much better off) to “7” (Very much less off).

Relationships with parents and friends before the age of 18 years old were investigated by asking participants about their satisfaction with their relationships with their mother, father, and friends. The three items were on a 5-point Likert scale from “1” (Very satisfied) to “6” (There is no such person).

Parental rule, monitoring and support at age 15 years old were examined using two items each (e.g., “My parent(s) set definite rules about what I was allowed to do at home”, “My parent(s) knew whom I was with in the evenings”, and “I could easily get warmth and caring from my mother and/or father”) on a 5-point Likert scale from “0” (Almost always) to “4” (Almost never) following previous studies (Foster, Gmel, & Mohler-Kuo, 2022; Marmet et al., 2018; Rougemont-Bücking, Grazioli, Daeppen, Gmel, & Studer, 2017).

Parental attitudes towards the consumption of alcohol, substances, and drugs when the respondent was approximately 15 years old were assessed using 6 items partially adapted from a previous study (Wood, Read, Mitchell, & Brand, 2004) with each item scored on a 4-point Likert scale from “0” (would not (did not) allow it) to “3” (would (did) approve it). Participants responded twice to the items to report maternal and paternal attitudes, respectively. Maternal and paternal attitude scores were averaged to compute a composite score.

Finally, family (i.e., mother, father, brothers/sisters) history of any mental disorder (i.e., psychiatric, alcohol or drug problems) was analyzed and coded as “0” (absence of family history of mental disorder) or “1” (its presence).

##### Individual factors

Personality traits measured at baseline were sensation-seeking, assessed using the Brief Sensation-Seeking Scale (BSSS) (Hoyle, Stephenson, Palmgreen, Pugzles Lorch, & Donohew, 2002) with eight items on a 5-point Likert scale from “1” (strongly disagree) to “5” (strongly agree), and aggression/hostility, sociability, and neuroticism/anxiety, evaluated using the Zuckermann–Kuhlmann Personality Questionnaire (Aluja et al., 2006) with each subscale made of ten true/false questions.

Civil status, number of children and partner pregnancy (no/yes), professional situation, and education were assessed at each time point to analyze whether they predicted change in group membership. Civil status was coded as single (“0” = single, widow, divorced, married but separated) or not (“1” = married, living together with my partner). Current professional status was coded as in education/employed (“0”), jobless/looking for a job (“1”), disability insurance/social security (“2”). Highest achieved education was explored using seven alternatives from secondary education (“0”) to bachelor-university (“6”).

#### Outcomes

Life satisfaction was examined using the Satisfaction with Life Scale (Diener, Emmons, Larsen, & Griffin, 1985) composed of five items rated on a 7-point Likert scale from “1” (strongly disagree) to “7” (strongly agree). Furthermore, negative life events during the last 12 months were assessed using 15 items originally designed to assess drug-use-related consequences, rated on a 5-point scale from “1” (never) to “5” (10 times or more often (Bucholz et al., 1994; Hesselbrock, Easton, Bucholz, Schuckit, & Hesselbrock, 1999; Wechsler, Davenport, Dowdall, Moeykens, & Castillo, 1994). These items included social and physical events and did not mention substance involvement (except the last one).

### Data analysis

Data were analyzed using IBM SPSS v. 25 and Mplus v. 8.6 (Muthén & Muthén, 1998). A latent transition analysis (LTA) framework (Collins & Lanza, 2009) was employed to identify latent profiles of substance and behavioral addiction symptoms and characterize their stability and change across three time points. As a preliminary step (Spurk, Hirschi, Wang, Valero, & Kauffeld, 2020), separate latent profile analyses (LPAs) were conducted for each wave to determine the optimal number of profiles at each time point. Multiple statistical fit parameters guided profile enumeration (Spurk et al., 2020; Zyberaj, Bakaç, & Seibel, 2022), including: (a) absolute and relative information criteria—Akaike Information Criterion (AIC; Akaike, 1987), Bayesian Information Criterion (BIC; Schwarz, 1978); (b) the Lo–Mendell–Rubin adjusted likelihood ratio test (Adj-LMR; Lo, Mendell, & Rubin, 2001), which compares the fit of a (*k*-1)-profile model with a *k*-profile model; and (c) entropy values, which quantify the overall ability of a mixture model to yield well-separated profiles. The number of profiles was determined by integrating statistical fit indices, theoretical interpretability, and parsimony (Spurk et al., 2020). A robust estimation strategy, i.e., maximum likelihood with robust standard errors (MLR; Muthén & Muthén, 1998), was applied to account for non-normal distributions (Vermunt & Magidson, 2002).

Following the identification of the optimal number of profiles at each time point, two LTAs models were estimated to verify whether the obtained measurement model was invariant across time (e.g., Zyberaj et al., 2022): an invariant LTA model (i.e., same correspondence between the observed indicators and latent profiles across the three time points) and a non-invariant LTA model (in which these parameters were free to vary across time). Model comparison was based on relative fit indices (AIC, BIC, and ssBIC), with lower values indicating better model fit. Afterwards, based on the invariant LTA solution, profiles that were consistently identified over time were interpreted and labelled using Welch's ANOVAs, by comparing mean levels of the substance-related indicators across profiles. Bonferroni's corrections were applied to account for multiple comparisons. Additionally, the prevalence (i.e., size) of latent profiles at each wave was calculated, and transition probability matrices were estimated to examine the probability of transitioning from a particular latent profile at time *t* to another latent profile at time *t* + 1 (i.e., logistic regression coefficient paths between the latent profiles; Collins & Lanza, 2009). Importantly, to further investigate factors influencing transitions, a main-effect LTA model was implemented to examine demographic and personality variables associated with transitioning between profiles over time. Results were reported as ORs with corresponding confidence intervals.

Lastly, to validate the retained profile solution across a range of predictors and outcomes (i.e., criterion-related validity; Spurk et al., 2020), participants were assigned to their most likely profile using modal class assignment (Woo, Jebb, Tay, & Parrigon, 2018). Predictors of latent profile membership at baseline (T1) were examined through multinomial logistic regression analyses, incorporating socio-demographic, parental, and personality variables. Odds ratios (ORs) and associated *p*-values were calculated to evaluate the relative likelihood of belonging to each profile. Moreover, two general linear models were estimated, testing whether T3 profile membership could predict mental health-related distal outcomes at T4 (i.e., life satisfaction and number of negative life events), while adjusting for their respective T3 levels (i.e., autoregressive effects; Spurk et al., 2020). Effect sizes were expressed as Cohen's *d*.

### Ethics

The Ethics Committee for Clinical Research of Lausanne University Medical School approved the original study (Protocol No. 15/07) (Gmel et al., 2013). The research in this paper did not require ethics board approval because it is a secondary analysis of anonymous data and only aggregated data are presented.

## Results

### Descriptive statistics and profile enumeration

Table S1 (Supplementary material) reports descriptive statistics and zero-order correlations among the variables included in the LTA models. Symptoms of addiction to alcohol (*r* range 0.41–0.52), tobacco (*r* range 0.57–0.66), cannabis (*r* range 0.54–0.71), video gaming (*r* range 0.40–0.56), and problematic gambling (*r* range 0.38–0.52) remained at least moderately stable over the years.

In the first step, five LPAs were conducted separately for each measurement point to identify the number of profiles that better describe the patterns observed in the data. Table 1 reports the absolute and relative fit indices of the measurement models. A three-profile solution was determined as the optimal number of profiles at each time point by integrating statistical fit values, classification accuracy (i.e., entropy), substantive interpretability, and content-related considerations (Spurk et al., 2020). Detailed information regarding the criteria and rationale for determining the optimal number of profiles at each time point is provided in the Supplementary material.

**Table 1.** Model fit and diagnostic criteria for LPA models with 1–5 profiles by survey wave

Model	#npar	Log-likelihood	AIC	BIC	Entropy	Adj LMR *p* value	Smallest class (*n*)
*Time 1*							
1	10	−24168.647	48357.294	48421.970	NA	NA	NA
2	16	−23221.271	46474.542	46578.024	0.721	<.001	599
3	22	−22638.630	45321.259	45463.546	0.762	.003	225
4	28	−22239.966	44535.931	44717.024	0.765	.057	112
5	34	−21900.844	43869.689	44089.586	0.775	.065	98
*Time 2*							
1	10	−30308.018	60636.036	60700.716	NA	NA	NA
2	16	−27594.117	55220.235	55323.723	0.993	<.001	281
3	22	−26052.837	52149.674	52291.970	0.995	<.001	113
4	28	−24917.023	49890.045	50071.149	0.966	<.001	110
5	34	−24100.999	48269.999	48489.911	0.968	.162	73
*Time 3*							
1	10	−27142.381	54304.762	54369.442	NA	NA	NA
2	16	−24168.430	48368.861	48472.349	0.995	<.001	259
3	22	−22967.958	45979.916	46122.212	0.993	.127	132
4	28	−22509.241	45074.482	45255.586	0.960	.002	132
5	34	−21318.688	42705.376	42925.288	0.978	.096	49

*Note*. The smallest class numbers are based on the most likely latent profile membership. Abbreviations: *NA*, not available; #npar, number of estimated free parameters.

### Interpretation, labeling, and predictors of latent profiles

Two LTAs were conducted to examine the longitudinal invariance of the parameters of the measurement model, one with invariance constraints applied across time (i.e., the same correspondence between the observed indicators and the latent profiles across time) and one without (i.e., the baseline model). Compared to the non-invariance model (AIC = 142785.639; BIC = 143264.272; ssBIC = 143029.127), the invariant one showed lower information criteria parameters (AIC = 142359.055; BIC = 142643.647; ssBIC = 142503.831), supporting measurement equivalence across time points and allowing us to examine the profile stability and transitions.

**Fig. 1.**
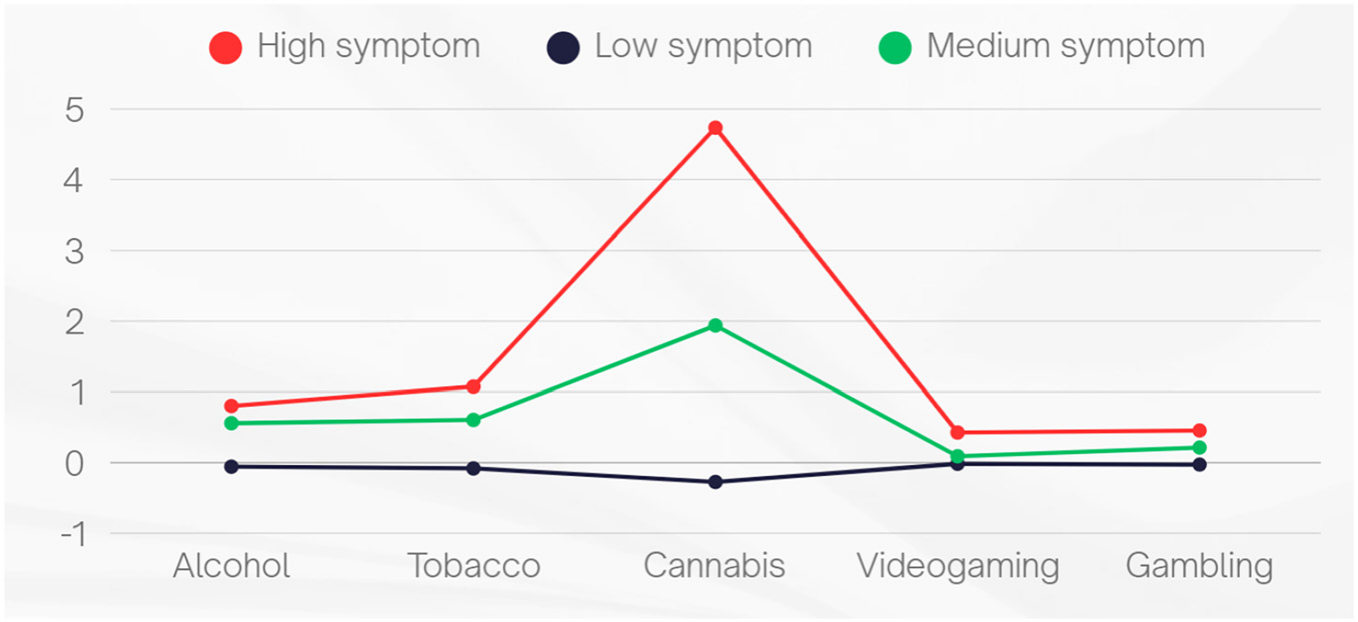
Shape and level of the *k* = 3 profiles of substance and behavioral addiction symptoms according to the invariant LTA model. *Notes*. Plotted values are expressed as standardized z-scores

Figure 1 depicts the results of the invariant LTA model using standardized mean scores. The majority of participants (92.1% at T1, 91.3% at T2, and 91.7% at T3) were classified in Profile 2 at each measurement point, which showed the most favorable pattern of substance and behavioral addiction symptoms and was thus labeled the *Low*-symptom profile. A smaller proportion (6.7% at T1, 6.1% at T2, and 5.4% at T3) belonged to Profile 3, characterized by a moderate severity of addiction symptoms and named the *Medium*-symptom profile, while the smallest group (1.2% at T1, 2.6% at T2, and 2.9% at T3) fell into Profile 1, which exhibited the least favorable pattern of addiction symptoms and was therefore labeled the *High*-symptom profile. The overall classification accuracy (i.e., entropy) was 0.975, showing that cases could be allocated to the correct latent profile with satisfactory certainty. Moreover, the average latent profile probability for the most likely profile was 0.920, 0.985, and 0.948 for High-, Low-, and Medium-symptom profiles, respectively, suggesting a very high degree of separation between the three profiles. To label profiles, we compared them to each other at T1 in terms of addiction symptoms dimensions using Welch's ANOVAs with post-hoc tests and Bonferroni's corrections (Table 2), with additional information described in the Supplementary material (Tables S2 and S3).

**Table 2.** Welch's ANOVAs testing differences in substance and behavioral addiction symptom dimensions across latent profiles at T1. Bonferroni's corrections were applied for post-hoc comparisons

Variable	*F* value (df)	Post-hoc	*η* ^2^
Alcohol_T1	37.33 (2, 101.97)**	High, Medium > Low	0.039
Tobacco_T1	84.42 (2, 101.18)**	High > Medium > Low	0.074
Cannabis_T1	2231.424 (2, 97.77)**	High > Medium > Low	0.857
Gaming_T1	4.26 (2, 105.14)*	High > Medium, Low	0.004
Gambling_T1	6.383 (2, 59.63)**	High, Medium > Low	0.009

*Note.* Participants were assigned to their most likely profile using modal class assignment. *p <.05, **p <.01.

To validate the latent profile solution, predictors of profile membership were examined (see Table S4 for descriptive statistics). More specifically, socio-demographic, parental, and personality variables were investigated in relation to the T1 profiles, with individuals assigned to their most likely latent profile using the modal class assignment. Multinomial logistic regression analyses were conducted to identify factors associated with membership in the High- and Medium-symptom profiles compared to the Low-symptom profile as the reference condition (see Table 3; Nagelkerke's *R*-squared = 0.142). Results indicated that higher scores on sensation seeking, neuroticism, parental attitudes toward substance use, poor parental monitoring, and poor relationships with parents and friends were significantly associated with increased odds of membership in the High-symptom profile compared to the Low-symptom profile (Table 3). Similarly, higher scores on sensation seeking, hostility, sociability, neuroticism, poor parental monitoring, and parental attitudes toward substance use, as well as reporting a family history of mental disorders, were associated with increased odds of membership in the Medium-symptom profile compared to the Low-symptom profile (Table 3). Note that demographic variables such as civil status, current professional status, number of children, and pregnancy status showed limited variability within the Medium- and High-symptom profiles (e.g., *n* ≤ 2 participants in categories such as 'married,' 'pregnant,' or 'having children'). Consequently, these variables were not included in the analyses.

**Table 3.** Multinomial logistic regression examining the association between socio-demographic, parental, and personality variables and T1 profiles (low symptom profile as a reference)

	Predictor	Estimate	*p*-value	OR
*“High” vs “Low”*	Sensation seeking	0.064	.014	1.066
	Hostility	0.056	.442	1.058
	Sociability	0.115	.148	1.122
	Neuroticism	0.203	.003	1.225
	Parents' financial situation	0.233	.133	1.262
	Relationships with parents and friends	0.231	.001	1.260
	Education	−0.028	.737	0.973
	Family history of mental disorders	0.524	.125	1.688
	Parental attitudes	0.836	.003	2.307
	Parental rule	−0.037	.643	0.963
	Parental monitoring	0.315	<.001	1.371
	Parental support	−0.166	.120	0.847
*“Medium” vs “Low”*	Sensation seeking	0.080	<.001	1.083
	Hostility	0.075	.016	1.078
	Sociability	0.112	.001	1.118
	Neuroticism	0.154	<.001	1.166
	Parents' financial situation	0.081	.229	1.084
	Relationships with parents and friends	0.045	.190	1.046
	Education	0.002	.962	1.002
	Family history of mental disorders	0.604	<.001	1.830
	Parental attitudes	0.308	.029	1.361
	Parental rule	0.068	.051	1.071
	Parental monitoring	0.143	<.001	1.154
	Parental support	0.010	.841	1.010

### Transition probabilities

Table 4 reports the transition probabilities of profiles from T1 to T2 and T2 to T3, i.e., the probability of transitioning from a specific profile at one time to all the other profiles at the subsequent time point. Most participants maintained their profiles, although transitions to other profiles also occurred.

**Table 4.** Transition probabilities based on the estimated invariant LTA model

	Time 2				Time 3		
Time 1	High	Low	Medium	Time 2	High	Low	Medium
High	0.494	0.345	0.151	High	0.408	0.260	0.332
Low	0.003	0.966	0.031	Low	0.008	0.969	0.024
Medium	0.247	0.285	0.469	Medium	0.184	0.416	0.400

In the case of T1–T2 transitions with High symptoms as the initial status, 49.4% of the members remained in the same profile, 34.5% of members were expected to move to the Low-symptom profile, and 15.1% to move to the Medium-symptom profile. The trend was slightly different for T2–T3 (40.8%, 26%, and 33.2%, respectively). The probability of transitioning from the High to the Medium symptom profile doubled, and those of transitioning from the High- to the Low-symptom profiles and remaining in the High-symptom profile decreased. Therefore, the probability of transitioning to a less severe profile of addiction symptoms increased overall.

In the case of T1–T2 transitions with Low symptoms as the initial status, most of the participants remained in the same profile (96.6%), 0.3% of the sample were expected to move to the High-symptom profile, and 3.1% to move to the Medium-symptom profile. The trend was nearly the same for T2–T3 (96.9%, 0.8%, and 2.4%, respectively).

Finally, the examination of the T1–T2 transitions with Medium symptoms as the initial status showed that 46.9% of the members remained in the same profile, 28.5% of members were expected to move to the Low-symptom profile, and 24.7% to move to the High-symptom profile. The trend substantially differed for T2–T3 (40%, 41.6%, and 18.4%, respectively) as the probability of transitioning from the Medium- to the Low-symptom profile increased, while the probability of transitioning from the Medium- to the High-symptom profile simultaneously decreased.

### Predictors of profile transition

To investigate whether inter-profile transitions of individuals were predicted by education, sensation seeking, hostility, sociability, and neuroticism, a main-effect LTA was estimated. ORs of the effects of predictors on transitioning are presented in Table 5, with stability (i.e., remaining in the same profile) employed as the reference condition. Education significantly and positively influenced the transition from the High profile to the Low profile, as well as from the Medium profile to the Low profile. Conversely, Education negatively affected the transition from the Low profile to both the High and Medium profiles. As the value of education increased by one unit, participants from the High and Medium profiles were more likely to transition into more adaptive profiles, whereas participants from the Low profile were less likely to transition into more maladaptive profiles.

**Table 5.** Odds ratios (ORs) of the effects of predictors on transition probabilities

	T2 Profile				T3 Profile		
T1 Profile	Medium	High	Low	T2 Profile	Medium	High	Low
**Education**							
Medium	Ref.	0.923	1.070	Medium	Ref.	0.935	1.101*
High	1.083	Ref.	1.159*	High	1.070	Ref.	1.177*
Low	0.935	0.863*	Ref.	Low	0.909*	0.849*	Ref.

**Sensation seeking**							
Medium	Ref.	0.974	0.907*	Medium	Ref.	0.997	0.926*
High	1.027	Ref.	0.931*	High	1.003	Ref.	0.929*
Low	1.103*	1.074*	Ref.	Low	1.080*	1.077*	Ref.

**Hostility**							
Medium	Ref.	1.149*	0.953	Medium	Ref.	0.956	0.899*
High	0.870*	Ref.	0.830*	High	1.046	Ref.	0.940
Low	1.049	1.205*	Ref.	Low	1.112*	1.063	Ref.

**Sociability**							
Medium	Ref.	1.012	0.949	Medium	Ref.	1.020	0.972
High	0.988	Ref.	0.938	High	0.981	Ref.	0.953
Low	1.054	1.066	Ref.	Low	1.029	1.049	Ref.

**Neuroticism**							
Medium	Ref.	1.125*	0.951	Medium	Ref.	1.109	0.998
High	0.889*	Ref.	0.845*	High	0.901	Ref.	0.900*
Low	1.051	1.183*	Ref.	Low	1.002	1.111*	Ref.

*Note.* Ref. is the comparison group for the multinomial logistic regressions. * *p* < .05.

When the value of sensation seeking increased by one unit, participants from the Low profile were more likely to transition into more maladaptive profiles, such as from the Low profile to the Medium and High profiles, whereas participants from the High and Medium profiles were less likely to transition into the Low profile. A similar pattern was found for hostility, with higher levels being associated with a higher likelihood of transitioning from adaptive to maladaptive profiles (e.g., from Low to Medium and High), and a lower likelihood of transitioning from maladaptive to adaptive profiles (e.g., from High to Medium). Furthermore, hostility positively predicted the transition from the Medium profile at T1 to the High profile at T2. Overall, the same pattern emerged for neuroticism: a one-unit increase was associated with participants from the Low profile being more likely to transition into maladaptive profiles (e.g., from Low to High), while participants from the maladaptive profiles were less likely to transition into adaptive profiles (e.g., from High to Low). As for hostility, neuroticism positively predicted the transition from the Medium profile at T1 to the High profile at T2. Lastly, sociability did not significantly affect transitions between T1 and T2 or between T2 and T3.

### Distal outcomes of profile membership

Finally, two general linear models were implemented to investigate whether profile membership at T3 could predict mental health-related distal outcomes at T4, while controlling for their respective baseline (T3) values. Results indicated that, compared to the Low-symptom profile, individuals in the High- (Cohen's *d* = −0.320, *p* < .001) and Medium- (Cohen's *d* = −0.153, *p* = .015) symptom profiles reported lower life satisfaction at T4 (overall *R*^2^ = 0.378). Similarly, compared to the Low-symptom profile, individuals in the High- (Cohen's *d* = 0.307, *p* < .001) and Medium- (Cohen's *d* = 0.219, *p* < .001) symptom profiles experienced a greater number of negative life events at T4 (overall *R*^2^ = 0.191).

## Discussion

### Addiction symptom profiles

In this study, we identified three latent profiles at baseline: a High-symptom profile showing high symptoms of all addictive behaviors; a Medium-symptom profile displaying moderate symptoms of addictive behaviors; and a Low-symptom profile reporting low symptoms of all addictive behaviors. Overall, our results confirm the co-occurrence of multiple addictive behaviors, in line with previous studies (Burleigh et al., 2022; Deleuze et al., 2015). However, unlike some prior studies utilizing LPA, we identified two profiles characterized by distinct severity of addictive behaviors. It is noteworthy that the two addictive behaviors that do not discriminate between the High and Medium profiles are alcohol consumption and gambling engagement, which are the most socially accepted behaviors in Western countries (Russell, Langham, & Hing, 2018; Sudhinaraset, Wigglesworth, & Takeuchi, 2016). From the syndrome model perspective (Shaffer et al., 2004), this data may be explained in terms of high social diffusion and, therefore, accessibility of these addictive behaviors. Notably, our data indicate that symptoms of nicotine dependence, cannabis use disorder, and gaming addiction differentiated the Medium and High profiles. This finding could be attributed to differences in opportunity and accessibility that shape the manifestation of addiction (Shaffer et al., 2004). Complementarily, aligning with the circle of reciprocity hypothesis (Burleigh et al., 2022) which posits that problematic behaviors may mutually exacerbate each other, our findings point to a close relationship between tobacco, cannabis, and gaming addictions, which may frequently co-occur during engagement episodes. For instance, South Korean adults who engage in both gaming and alcohol use report higher tobacco consumption than those involved in only one of these behaviors (Na, Lee, Choi, & Kim, 2017).

According to the strength of effect sizes (i.e., *η*^2^), cannabis use emerged as a key discriminator between the three profiles. Since our analysis did not examine engagement with other illicit substances, we cannot clarify whether individuals in this profile also used other illicit drugs such as heroin or cocaine, which have been identified as significant profile discriminators in prior research (Deleuze et al., 2015).

### Addiction profile and vulnerability factors

Identifying profiles of co-occurring addictive behaviors alone does not clarify the underlying mechanisms driving this co-occurrence, which may be linked to shared vulnerability factors and/or reciprocal exacerbation effects among behaviors, according to the transdiagnostic syndrome model of addiction (Shaffer et al., 2004) and the circle of reciprocity hypothesis (Burleigh et al., 2022), respectively. Both the Medium- and High-symptom profiles significantly differ from the Low-symptom profile in personality traits, particularly sensation-seeking and neuroticism, as well as parental attitudes toward substance use. However, each symptomatic profile also exhibits distinct risk factors. The High-symptom profile was associated with poor relationships with friends and parents when participants were 18 years old, while the Medium profile was distinguished by high hostility, high sociability, low parental rule-setting when participants were 15 years old, and the reporting of a family history of mental disorders. These results align - but also extend - Shaffer et al.'s (2004) hypothesis that personality traits represent a common basis for engagement in addictive behaviors. Sensation-seeking and neuroticism have been widely recognized as transdiagnostic factors in addiction (Shaffer et al., 2018), particularly in relation to positive urgency and emotional dysregulation (e.g., Amendola, Spensieri, Guidetti, & Cerutti, 2019; Rogier, Colombi, & Velotti, 2022; Velotti, Rogier, Beomonte Zobel, & Billieux, 2021). Hostility and sociability characterizing the Medium profile suggest that individuals in this group may be navigating a critical life stage involving identity development and social integration (de Moor, Nelemans, Becht, Meeus, & Branje, 2023). In contrast, the High profile showing poorer interpersonal relationships when profile members were 18 years old points to the importance of socio-relational aspects in addictive disorders (Fairbairn et al., 2018; Skeer, McCormick, Normand, Buka, & Gilman, 2009; Spohr, Livingston, Taxman, & Walters, 2019; Tan, Mun, Nguyen, & Walters, 2021; Ten Have et al., 2019; Vrijen, Wiertsema, Ackermans, van der Ploeg, & Kretschmer, 2021).

### Addiction profile trajectories, education, and personality

Regarding stability, individuals with low or no symptoms of addictive disorders demonstrated high stability over time, suggesting that chronic addiction is less likely to emerge during young adulthood (Chen, Storr, & Anthony, 2009). Furthermore, 40–50% of individuals in the two symptomatic profiles remained in their respective categories over time, with transitions following distinct patterns. Over successive measurement waves, the proportion of individuals transitioning from the Medium to the High profile decreased. In contrast, transitions from the High to the Medium profile exhibited an inverse trend, increasing over time. Additionally, individuals with high symptoms were increasingly unlikely to transition into the Low profile over time, whereas the probability of transitioning into this profile increased for those from the Medium profile. These findings support the syndrome model by indicating that individuals with more severe addictive behavior struggle to reduce or interrupt their engagement over time, potentially due to increased vulnerability resulting from addiction-related psychosocial consequences. This aligns with previous research showing that addictive disorders are likely to be chronic or long-term conditions (Amendola, Hengartner, Ajdacic-Gross, Angst, & Rössler, 2022; Fleury et al., 2016). However, our analysis also highlighted that some individuals, particularly those of the Medium profile, have a significant probability of interrupting addictive behaviors. This stresses the notion that “addiction is not destiny,” challenging prior studies which reported very high profile stability at one-year follow-up (e.g., Sussman et al., 2015).

Our results clarified the mechanisms facilitating transitions from one class to another. First, the role of personality traits such as sensation seeking, hostility, and neuroticism appears relevant in explaining why some individuals transit from more adaptive profiles to less adaptive ones and should be interpreted in light of the potential interaction between these personality traits and contextual changes during the life course. Indeed, early adulthood is characterized by pronounced changes such as challenges associated with entry into the labor or academic system as well as changes in the interpersonal entourage (e.g., Azmitia, Syed, & Radmacher, 2013; Zucker, Hicks, & Heitzeg, 2016). Our results suggest that individuals with high levels of sensation-seeking could struggle to reach satisfaction with life characterized by a predictable routine. The difficulty in tolerating boredom in everyday life could exacerbate the engagement in addictive behaviors or motivate their onset. Moreover, individuals characterized by a negative view of the interpersonal environment - i.e., high in hostility - were more likely to transit to more maladaptive profiles. This finding is in line with those from a previous study on profiles of problematic technology use (Amendola, Spensieri, Biuso, & Cerutti, 2020) and was discussed as evidence on the role of the *agenda protection system* (Harkness, Reynolds, & Lilienfeld, 2014) in motivating individuals to prioritize their desires, giving rise to emotional or behavioral reactions of submissiveness, passivity, irritation, anger and aggression. In this perspective, addictive conduct would represent a different way of fulfilling previously frustrated needs. Also, the role of neuroticism in predicting transition between profiles suggests that the risk of developing addictive disorders increases with time in individuals with poor emotional stability. This may be due to the new stressors characterizing early adulthood that, with time, exert a growing pressure exacerbating individuals' vulnerabilities, which may turn to addictive substances and behaviors to self-medicate from psychological suffering related to negative and painful emotions, self-esteem, and social relationships, to manage self-regulation problems and obtain emotional relief (Khantzian, 1997, 2012).

Lastly, we found that higher levels of education predict an increased probability of transitioning from less adaptive to more adaptive profiles and decrease the probability of transitioning from more adaptive to less adaptive profiles. Several explanations for this result point to the role played by the external context. First, reduced opportunity and accessibility to addictive behaviors characterizing highly qualified work environments and academic contexts may play a role. For instance, individuals with a higher level of education have a higher probability of engaging in university courses that pose limitations in terms of time that can be devoted to addictive behaviors. Second, school problems are often associated with a negative social context and a higher probability of becoming part of a deviant peer group (Rudasill, Niehaus, Crockett, & Rakes, 2014), which, in turn, is a widely known risk factor for engagement in multiple addictive behaviors (Kirisci, Tarter, Mezzich, & Vanyukov, 2007).

### Addiction profile, life satisfaction, and adverse life events

The third objective of our study was related to the analysis of potential consequences of symptoms of addiction and, specifically, profile membership. We observed that individuals belonging to the Medium- and High-symptom profiles were more likely to experience negative life events as well as decreased life satisfaction over time, compared to those in the Low-symptom profile. This is in line with previous evidence showing a close interrelation between symptoms of substance use disorder and life satisfaction (Moe, Erga, Bjornestad, & Dettweiler, 2024) as well as bidirectional longitudinal associations between symptoms of gaming disorder and life satisfaction (Amendola, Bernath, Presaghi, Waller, & Hengartner, 2025). Worth noting, we found that symptoms of addictive disorders predicted adverse life events at subsequent follow-up after controlling for adverse life events at the previous wave. On the one hand, addictive disorders may indeed expose individuals to highly risky situations, as shown by previous research (e.g., Estévez-Lamorte, Foster, Gmel, & Mohler-Kuo, 2021). On the other hand, adverse life events are associated with subsequent psychopathology in adolescence and young adulthood (Asselmann, Wittchen, Lieb, Höfler, & Beesdo-Baum, 2016) and may thus configure and/or maintain the individual in a vicious circle of addiction and negative outcomes.

### Strengths and limitations of the study

The population-based design and the longitudinal data collection covering 10 years constitute valuable strengths of the present analysis, ensuring generalizability of the findings to the population of young Swiss men. Therefore, this data provided a unique opportunity to analyze latent profile membership and trajectories in symptoms of substance and behavioral addictions. Similarly, it enabled us to examine the role of personality traits in predicting profile membership and the association between profile membership and subsequent physical and mental health, life satisfaction, and negative life events. Nevertheless, certain limitations need to be considered when interpreting the study findings. The first limitation is the use of self-reports rather than clinical diagnostic interviews to ascertain the presence of addictive disorders. Thus, our findings have to be interpreted as exploring a condition at risk of addictive disorders. Related to this aspect, it is possible that social desirability influenced participants' reporting about their substance use and involvement in excessive behaviors. Second, our findings might not be generalized and valid if applied to young Swiss females and other age groups of the population. Third, we could not include some relevant sociodemographic variables as originally planned. As described in the results section, the effect of civil status, current professional status, number of children, and pregnancy status was not tested, as these variables showed limited occurrence across the Medium- and High-symptom profiles.

## Conclusions

Our findings suggest that individuals engaging in multiple addictive behaviors, particularly those involving co-occurring use of tobacco, cannabis, and gaming, warrant special clinical attention, as they are likely to exhibit higher severity across all addiction types. The finding that a consistent proportion of individuals with more severe addictive behaviors struggle to reduce or interrupt their engagement over time points to the importance of addiction-related psychosocial consequences, maintaining the individuals in a vicious circle. Future research that focuses on the analysis of risk factors specific to individuals showing high-symptom stability over time would be highly valuable. These individuals might benefit from meaningful interventions targeting psychosocial risk factors (e.g., poor interpersonal relationships) or individual factors (e.g., neuroticism and sensation seeking) and providing new healthy opportunities for needs satisfaction (e.g., involvement in social activities and support, education, and work experiences).

## Supplementary material

## Data Availability

The data used in this article may be requested at https://www.c-surf.ch/en/25.html following the application procedure for research projects, and via the open research repository Zenodo (Gmel et al., 2021). The Full Research Proposal conceptualizing the analysis conducted in this study – submitted to the C-SURF Scientific Board on April 4^th^, 2024, to inform about the plan for this secondary analysis and request access to the data – can be accessed at https://osf.io/agwcp.
