# Supplementary material for: Co-occurrence of symptoms of substance and behavioral addictions over time: A secondary analysis of longitudinal data from the Cohort Study on Substance Use Risk Factors
Source: J Behav Addict. 2025 Nov 24;14(4):1503–16. doi: 10.1556/2006.2025.00088 (PMC12767602; doi:10.1556/2006.2025.00088)
Supplement: Supplementary file 1 [file jba-14-1503-s001.pdf]

Zagaria, A. et al.: Co-occurrence of symptoms of substance and behavioral addictions over time: A secondary analysis of longitudinal data from the Cohort Study on Substance Use Risk Factors.

https://doi.org/10.1556/2006.2025.00088

## Supplementary material

**Table S1.** Descriptive statistics and zero-order correlations among modelled variables representing substance and behavioral addiction symptoms.

| Variable | M (SD) | 1 | 2 | 3 | 4 | 5 | 6 | 7 | 8 | 9 | 10 | 11 | 12 | 13 | 14 | 15 |
|---|---|---|---|---|---|---|---|---|---|---|---|---|---|---|---|---|
| 1.Alcohol_T1 | 1.35 (1.83) | 1 | | | | | | | | | | | | | | |
| 2.Alcohol_T2 | 1.28 (1.72) | .52* | 1 | | | | | | | | | | | | | |
| 3.Alcohol_T3 | 1.29 (1.69) | .41* | .51* | 1 | | | | | | | | | | | | |
| 4.Tobacco_T1 | 1.02 (1.53) | .12* | .07* | .06* | 1 | | | | | | | | | | | |
| 5.Tobacco_T2 | 0.70 (1.32) | .15* | .16* | .12* | .66* | 1 | | | | | | | | | | |
| 6.Tobacco_T3 | 1.03 (1.54) | .06* | .06* | .08* | .57* | .64* | 1 | | | | | | | | | |
| 7.Cannabis_T1 | 3.02 (5.95) | .18* | .11* | .14 | .26* | .25* | .19* | 1 | | | | | | | | |
| 8.Cannabis_T2 | 1.13 (4.01) | .17* | .20* | .20* | .22* | .28* | .20* | .71* | 1 | | | | | | | |
| 9.Cannabis_T3 | 1.39 (4.42) | .12* | .14* | .20* | .19* | .25* | .22* | .54* | .63* | 1 | | | | | | |
| 10.Videogaming_T1 | 11.61 (4.87) | .11* | .08* | .08* | .04* | .04* | .06* | .04 | .05* | .04* | 1 | | | | | |
| 11.Videogaming_T2 | 11.16 (4.56) | .05* | .09* | .06* | .05* | .04* | .05* | .01 | .03* | .03* | .56* | 1 | | | | |
| 12.Videogaming_T3 | 10.87 (4.33) | .06* | .04* | .09* | .07* | .07* | .15* | .09* | .07* | .14* | .40* | .46* | 1 | | | |
| 13.Gambling_T1 | 0.72 (0.60) | .13* | .07* | .09* | .12* | .10* | .06* | .10* | .06* | .04* | .09* | .08* | .07* | 1 | | |
| 14.Gambling_T2 | 0.69 (0.61) | .12* | .09* | .11* | .12* | .13* | .07* | .04 | .05* | .06* | .09* | .26* | .12* | .52* | 1 | |
| 15.Gambling_T3 | 0.45 (0.60) | .11* | .10* | .17* | .12* | .12* | .07* | .06 | .06* | .11* | .05* | .10* | .17* | .38* | .41* | 1 |

*Note*. * $p < .05$.

**Additional information about profiles enumeration**

At T1, while the interpretation of information criteria was rather inconclusive (i.e., they tended to decrease as the number of profiles increased), the three-profile solution was retained based on the significant LMR (p) value, suggesting that the 3-profile model fitted the data significantly better than a model with one less profile. In contrast, adding a fourth profile did not result in a statistically significant improvement in model fit. Moreover, no substantial improvement in entropy was observed in the four- and five-profile solutions, supporting the three profiles as the most parsimonious and interpretable solution.

At T2, the LMR (p) value suggested a four-profile solution as the most plausible model; however, this solution was associated with a reduction in entropy compared to the three-profile solution. This indicates a potential loss of classification accuracy and interpretability with the inclusion of an additional profile. Therefore, the three-profile solution was retained at T2, ensuring consistency with T1 and preserving the balance between interpretability and parsimony.

At T3, the LMR (p) value and entropy values favored a two-profile solution. However, this solution was less informative, as it simply dichotomized participants into high and low levels of substance use, offering limited differentiation and interpretative value. Consistent with the methodological literature, such solutions may be deprioritized in favor of those offering substantive interpretations of much theoretical interest (e.g., Spurk et al., 2020). Therefore, by integrating statistical fit values, substantive interpretability, and content-related considerations (Spurk et al., 2020), the three-profile solution was retained at T3. This solution offered comparable classification accuracy (i.e., entropy) to the two-profile solution while maintaining consistency with the profiles identified at T1 and T2.

**Additional information about the labeling of profiles**

ANOVAs revealed significant differences among the profiles across all variables (see in-text Table 2). Raw (unstandardized) means of addiction symptoms across profiles are reported below in Table S2. Specifically, for Alcohol, Tobacco, Cannabis, and Gambling, the Low symptom profile consistently scored significantly lower than both the High and Medium symptom profiles. For these variables, no significant differences were observed between the High and Medium symptom profiles, except for Tobacco and Cannabis, where scores of the High profile were significantly higher than those of the Medium profile. In the case of Gaming, the High profile scored significantly higher than both the Low and Medium profiles, with no significant differences between the latter two profiles. In Table S3, we considered items and questionnaire scores to inform the interpretation of symptoms experienced by participants belonging to the High profile.

**Table S2**. Raw (unstandardized) means of addiction symptom dimensions across profiles.

| Variable | High M (SD) | Low M (SD) | Medium M (SD) |
|---|---|---|---|
| Alcohol_T1 | 3.17 (3.47) | 1.25 (1.69) | 2.56 (2.69) |
| Tobacco_T1 | 3.22 (1.76) | 0.88 (1.43) | 2.02 (1.75) |
| Cannabis_T1 | 28.23 (4.15) | 0.95 (1.82) | 14.06 (4.00) |
| Gaming_T1 | 14.82 (7.90) | 11.56 (4.82) | 11.84 (4.92) |
| Gambling_T1 | 1.01 (1.04) | 0.70 (0.58) | 0.89 (0.77) |

*Note*. Participants were assigned to their most likely profile using modal class assignment.

**Table S3.** Questionnaire and items scoring for profile interpretation.

| Questionnaire | Item scoring | Range of possible total score | Mean score interpretation (examples) of the High symptom profile |
|---|---|---|---|
| Alcohol Dependence and Abuse criteria- Harvard School of Public Health College Alcohol Study | 12 symptoms of AUD with a yes/no format response | 0 - 12 | Three symptoms |
| Cannabis Use Disorder Identification Test | 10 items from 0 “never” to 4 “daily” | 0 - 40 | Near all symptoms reported "weekly" |
| Fagerström Test for Nicotine Dependence | Four questions with a yes/no format, plus one question scored from 0 to 3 | 0 - 7 | Three dichotomous items, or reporting smoking the first cigarette within 5 minutes after waking up |
| Game Addiction Scale | 7 items from 1 “never” to 5 “very often” | 7 - 35 | All symptoms "rarely", or "sometimes" to four items (polythetic format) |
| Problematic gambling symptoms (composite score) | 7 items about frequency of gambling behaviors from 0 “never” to 1 “daily”, and 1 item with a yes/no format that was weighted | 0 - 8 | One gambling behavior every day, or problems caused by gambling, or four different gambling behaviors a few times per year |

**Table S4**. Descriptive statistics of socio-demographic, parental, and personality variables (at baseline) included in multinomial logistic regressions.

| Variable | Mean (SD) / % |
|---|---|
| Sensation seeking | 24.35 (6.87) |
| Hostility | 4.12 (2.22) |
| Sociability | 5.85 (2.25) |
| Neuroticism | 1.96 (1.99) |
| Parents' financial situation | 3.60 (0.99) |
| Relationships with parents and friends | 5.31 (2.19) |
| Education | 2.75 (1.99) |
| Family history of mental disorders | 66.8 |
| Parental attitudes | 0.38 (0.44) |
| Parental rule | 4.47 (2.02) |
| Parental monitoring | 3.70 (1.91) |
| Parental support | 3.11 (1.54) |

*Abbreviations.* SD, standard deviation.

## References

Spurk, D., Hirschi, A., Wang, M., Valero, D., & Kauffeld, S. (2020). Latent profile analysis: A review and "how to" guide of its application within vocational behavior research. *Journal of Vocational Behavior*, *120*, 103445. https://doi.org/10.1016/j.jvb.2020.103445
